# Sustainable Food Security Through Nanotechnology-Based Seed Priming in Rice and Wheat

**DOI:** 10.3390/foods15152595

**Published:** 2026-07-24

**Authors:** Anuj Sharma, Vaibhav Sharma, Kumud Kant Awasthi, Mahipal Singh Sankhla, Ruhani Sharma, Anjali Awasthi, Sudhakar Srivastava, Garima Awasthi, Theodoros Varzakas

**Affiliations:** 1 Department of Forensic Science, Parul Institute of Applied Sciences, Parul University, Vadodara 391760, India; anujs0353@gmail.com; 2 Department of Medical Elementology and Toxicology, Jamia Hamdard [Deemed to be University], New Delhi 110062, India; vaibhav.s4n6@gmail.com; 3 School of Basic and Applied Sciences, K.R. Mangalam University, Gurugram 122103, India; kkantawasthi@gmail.com (K.K.A.); mahi4n6@gmail.com (M.S.S.); 4 Department of Zoology, University of Rajasthan, Jaipur 302004, India; ruhanisharma2206@gmail.com (R.S.); anjkam.awasthi@gmail.com (A.A.); 5 Institute of Environment & Sustainable Development, Banaras Hindu University, Varanasi 221005, India; sudhakar.srivastava@gmail.com; 6 Department of Food Science and Technology, University of the Peloponnese, 22100 Tripoli, Greece

**Keywords:** nano-priming, food security, rice, wheat, bibliometric analysis, nanotechnology, seed germination, abiotic stress

## Abstract

Nano-priming has emerged as a novel technology for improving seed germination, vigour, and stress tolerance, and enhancing nutrient uptake in cereal grains, especially rice and wheat. This study presents a bibliometric analysis of 6302 publications obtained by a Boolean search query from the Scopus database, executed in November 2025. The dataset was further refined by using strict inclusion–exclusion criteria and mapped for the intellectual, geographic, and collaborative structure of the research on the topic under study at the global level. Country-level research productivity, subject-area distribution, annual publication trajectories, source-level publication patterns, and co-authorship networks are part of this study. The analysis revealed a highly skewed global publication distribution dominated by China, India, and Pakistan, which are major global hubs for nanotechnology-assisted seed treatment research, on the nano-priming of seeds. Agricultural sciences, environmental sciences, materials science, and nanotechnology emerged as dominant interdisciplinary contributors to nano-priming research. Annual publication trends continue to show an exponential rise since 2013, driven by growing interests in nanotechnology-enabled crop improvement. Co-authorship analysis revealed dense collaborative clusters centred in South and East Asia, interconnected through key bridging authors. The bibliometric evaluation, together with evidence-based synthesis of experimental studies, highlighted zinc oxide, titanium dioxide, silver, iron oxide, chitosan, and carbon-based nanomaterials as the main hotspots driving physiological enhancement in rice and wheat through enzymatic activation, nutrient uptake, and stress-resilience pathways. Nanotechnology-based seed treatments in rice and wheat offer a promising and sustainable approach to enhance crop productivity, stress tolerance, nutrient-use efficiency, and global food security under changing environmental conditions.

## 1. Introduction

A pre-sowing procedure known as “seed priming” modifies the seed’s physiology to enable faster germination [1]. It enhances seed germination and plant stress tolerance through the increased tolerance of plants to biotic and abiotic stressors [2]. In other words, priming is a pre-seed treatment through conventional techniques such as coating and pre-soaking before planting. In the pre-germination stage, other methods of seed hydration are used either with or without aeration, but the seeds are re-dried again to the conventional moisture content typical of common handling procedures like planting, packing, and preservation.

Halo-priming, hydro-conditioning, osmo-priming, hormonal priming, bio-priming, magneto priming, matriconditioning, and nano-priming reduce seed dormancy with the use of salt solutions, water, osmotic agents, plant hormone solutions, beneficial microbe solutions, exposure to a magnetic field, a solution containing a solid carrier, and solutions containing NPs, respectively [3,4]. Hydro-priming, osmo-priming, hormonal priming, nutri-priming, on-farm priming, bio-priming, and nano-priming are modern seed enhancement approaches that improve germination, stress tolerance, and crop performance [5]. Priming agents need to be tailored to the characteristics and potentials of each crop species. For practical agricultural yields, nano-priming has been considered more promising when compared to conventional priming techniques [6]. Nano-priming typically involves engineered nanoparticles generally within the nanoscale range [<100 nm], although aggregation behaviour may influence effective particle size [7]. It has been reported that nano-priming may stimulate seed germination and seedling vigour in a variety of crops [7,8,9].

The sustainability of agricultural systems has been thoroughly assessed by frameworks, with emphasis on rice cultivation [10]. Hence, economic, environmental, social, and governance aspects of sustainability are being assessed with the aid of these frameworks using specific indicators for each dimension [10]. Different methodologies distinguish the scale of application, selected indicators, and level of stakeholder engagement [11]. Operational and resource-use efficiency are improved by the adoption of agroecological practices, and this takes place by replacing harmful inputs with eco-friendly alternatives and designing sustainable agricultural ecosystems [12]. Environmentally sustainable and productive farming systems are practiced by spatial bioengineering, nanotechnology, and soil management [13]. The importance of developing comprehensive diagnostic tools to reduce costs and minimise environmental impact is emphasised by means of integrated environmental and economic assessments of crops like maize and rapeseed [14].

Nanoparticles (NPs) are offered as innovative solutions to boost agricultural productivity while maintaining ecological balance, thus showing nanotechnology’s role in addressing these challenges. Hence, seed priming for improved germination rates by up to 30%, nanosensors for precise monitoring of environmental and crop health, and smart nutrient delivery systems that can increase nutrient uptake efficiency by 50% constitute such significant practices [15,16,17,18,19,20]. Nano-priming provides protection for seeds during storage, improvements in germination, germination synchronisation, and plant growth, and increases the resistance of crops to abiotic or biotic stress conditions, thus helping to reduce the required quantities of pesticides and fertilisers [21,22]. New studies showed that seed nano-priming can activate different genes during germination, especially those related to plant stress resistance [23,24,25,26]. Studies on the use of nanotechnology for seed priming have already shown promising results [23,26,27,28,29]. Many nanoparticles possess antimicrobial capabilities, and can load antimicrobial agents; hence, seed nano-priming also can be used for seed protection [6,27,30,31]. In addition, biofortification of seeds to promote an increase in food quality and production can be achieved by nano-priming [22,32].

This may be one of the most successful treatments for overcoming dormancy and enhancing seed germination in upland boreal forest species, and indeed may have other applications in forest reclamation, such as nano-priming [33]. Seeds and plants are also influenced by nanoparticles, according to recent research [5,34,35,36]. Some nanoparticles cause deleterious effects, such as inhibition of germination or phytotoxicity at the seedling stage. On the other hand, other nanoparticles may act as elicitors via intracellular signalling pathways, which may affect seed metabolism, including seedling vigour and plant growth. Physical-chemical characteristics of the nanoparticles, that is, size and concentration, as well as the zeta potential, affect biological responses [35,37] and have significant effects on the absorption and transportation process in plants. Thus, smaller nanoparticles can move through biological barriers more successfully compared to their larger counterparts [38,39,40,41]. The nanoparticles’ surface charge is also crucial. The leaves can absorb positively or negatively charged nanoparticles and move them to the roots. However, the roots only absorb negatively charged nanoparticles. Mucilage is produced in response to positive charges, preventing plants from absorbing them [42,43,44].

This article presents an in-depth bibliometric analysis to delineate the intellectual and collaborative landscape of nano-priming research. Publication output at the country level was analysed to determine the dominant contributors and to identify emerging research hubs. The disciplinary distribution of the publications mapped using subject classifications showed that agricultural sciences, environmental sciences, materials science, nanotechnology, and biotechnology were integrated in an interdisciplinary fashion. Temporal trends in annual publication output were analysed to assess the developmental trajectory and research momentum of the field. Source-level metrics were used to determine high-impact journals and core publication venues that have shaped the discourse. In addition, co-authorship network analysis was employed to investigate the structure and intensity of scientific collaboration across authors, institutions, and countries and therefore test key nodes and clusters in the global research ecosystem.

Nano-enabled seed treatments have shown significant potential in enhancing seed germination, nutrient uptake, stress tolerance, disease resistance, and overall crop performance while reducing excessive dependence on chemical fertilisers and pesticides. Research in this area has advanced along two largely separate tracks: bibliometric studies have mapped the growth, geography, and thematic structure of nano-priming research, whereas mechanistic and experimental studies have examined how nanomaterials act at the seed and seedling level, yet the two bodies of work have rarely been brought together. Consequently, it remains unclear how the quantitative trends emerging from the global research landscape on the dominant nanoparticle classes, the geographic concentration of output, and the shifting thematic emphasis map onto the biological mechanisms and dose–response behaviour that ultimately govern field performance and food-security outcomes in rice and wheat. The present review addresses this gap by explicitly integrating a bibliometric analysis of the global literature with a mechanistic synthesis of experimental evidence, so that the structure of the research field is interpreted in light of the biological processes it is intended to explain.

Accordingly, the specific objectives of this review are fourfold: (i) to characterise the global research landscape of nanotechnology-based seed priming in rice and wheat publication growth, leading countries and journals, collaboration structure, and dominant nanoparticle classes using quantitative bibliometric indicators; (ii) to translate these bibliometric patterns into a mechanistic synthesis, linking the most active research themes to the biological processes (reactive oxygen species regulation, hormonal signalling, nutrient and water transport, membrane and antioxidant responses, and transcriptional control) through which nano-priming influences germination, vigour, and stress tolerance; (iii) to critically appraise the experimental literature, including study quality, reproducibility, dose–response and hormetic behaviour, and the laboratory-to-field translation gap; and (iv) to identify the principal knowledge gaps and outline the future research and safe-and-sustainable-by-design priorities needed to move nano-priming from proof-of-concept toward scalable contributions to sustainable food security.

## 2. Methodology

### 2.1. Data Source and Search Strategy

The bibliometric dataset for this study was retrieved from the Scopus database, one of the most comprehensive and authoritative repositories for peer-reviewed scientific literature. An advanced search using a specifically developed Boolean search string in November 2025 assured that all relevant publications addressing nanotechnology-assisted seed treatment and its applications in rice and wheat were included. The query combined synonymous and related terms for nano-priming, nanotechnology-based treatments, and nanomaterials with crop-specific descriptors. The final Boolean string used was [“seed priming” OR “nano priming” OR “nanopriming” OR “nano-priming” OR “nanoparticle priming” OR “nanotechnology priming” OR “nano-enabled priming” OR “nano treatment” OR “nano-fertiliser priming” OR “nanofertiliser” OR “nanomaterials” OR “nanoparticles” OR “nano-agriculture” OR “nano-enabled seed treatment”] AND [“rice” OR “Oryza sativa” OR “paddy” OR “wheat” OR “*Triticum aestivum*”].

The search string was developed iteratively rather than applied in a single step. An initial core string built around the terms “seed priming” and “nano-priming” was executed in Scopus, and the retrieved records were inspected at the title and keyword levels to identify recurrent synonyms, hyphenation variants, and related descriptors that the core string had failed to capture (for example “nanopriming”, “nano-enabled seed treatment”, and “nanofertiliser”). These terms were incorporated in successive iterations, and crop descriptors were similarly expanded to include both common and binomial names (rice/Oryza sativa/paddy; wheat/Triticum aestivum). Each iteration was checked against a small set of benchmark publications known a priori to be relevant to nano-priming in rice and wheat, and the string was considered final when all benchmark records were returned and further term additions yielded only marginal or thematically irrelevant gains. This procedure constituted pragmatic validation rather than quantitative optimisation: no formal sensitivity analysis (for example, systematic comparison of recall and precision across alternative string variants, or a comparative benchmark against an independently compiled reference set) was performed, and the absence of such an analysis is acknowledged as a limitation of the search strategy.

There are some limitations associated with this study. First, the bibliometric analysis was performed only for publications listed in the Scopus database, and other databases might contain some relevant information that was not captured by the present bibliometric analysis, including Web of Science, PubMed, and regional databases. Second, the publication languages included in the analysis were restricted to English only, and this may have some linguistic bias. Moreover, bibliometric measures are affected by the quality of the database indexing process as well as the keyword selection approach.

### 2.2. Inclusion and Exclusion Criteria

Inclusion and exclusion criteria were systematically set to refine the dataset for bibliometric analysis. The inclusion criteria emphasised that the publications had to: [i] explicitly deal with nanotechnology-assisted seed treatment, nano-priming, nanotechnology-based seed treatment, or nanoparticle-mediated enhancement of seed physiology; [ii] pertain to rice [Oryza sativa] or wheat [Triticum aestivum]; and [iii] be accessible within the Scopus database at the time of the November search. Types of articles considered in this study include both experimental and review papers to have a wide representation of the research activity, trends, and scientific discourse. Publications were excluded if they: [i] did not involve either nanotechnology-assisted seed treatment or nanotechnology-based treatments even though they had appeared in the initial keyword search; [ii] did not focus on rice or wheat although general crop terms may have been used; [iii] were conference abstracts, editorials, book chapters without bibliometric relevance, or inaccessible due to missing metadata; or [iv] were duplicates of already existing records. These criteria ensured that the final dataset accurately represented the global research landscape about nanotechnology-assisted seed treatment in rice and wheat.

The initial Scopus search retrieved 9127 records related to nanotechnology-assisted seed treatment and nanotechnology-based seed treatments. After removal of duplicates, non-English records, conference abstracts, editorials, and records lacking direct relevance to nanotechnology-assisted seed treatment in rice and wheat, based on database indexing and metadata classification, a final dataset of 6302 publications was retained for bibliometric analysis.

**Figure 1:**
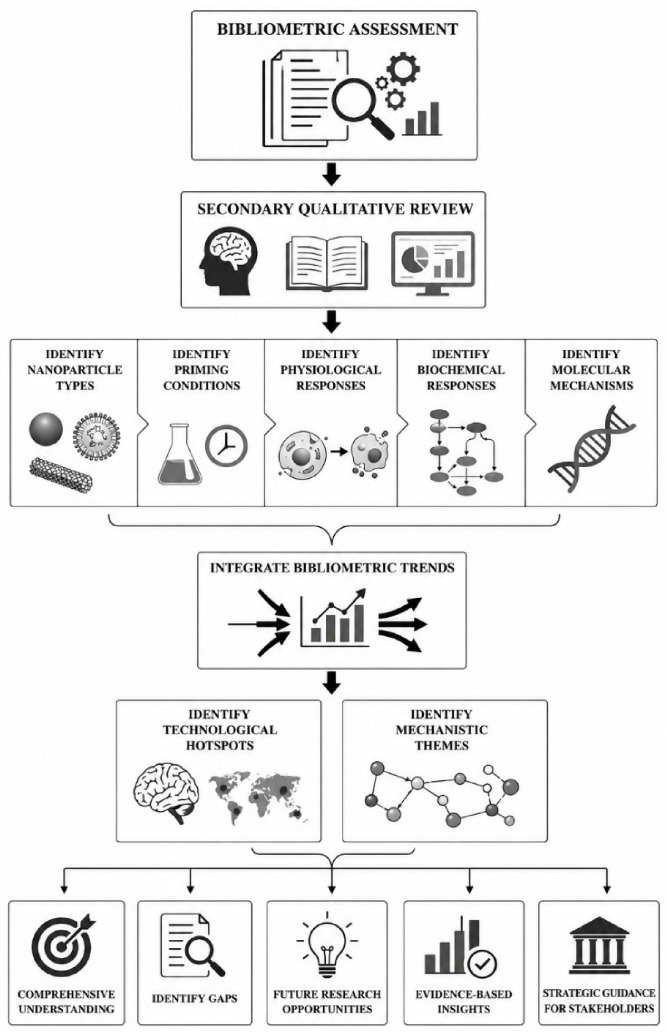
Flow diagram of the extraction and synthesis of experimental sets within the discipline of seed nano-priming.

To ensure transparency and reproducibility of the screening process, records were processed through sequential, clearly defined stages summarised in the flow diagram (Figure 1). After the initial retrieval of the 9127 records, exact and near-duplicate entries were removed automatically within Scopus and the reference-management workflow; non-English records and ineligible document types (conference abstracts, errata, and editorial or note items lacking bibliometric metadata) were then excluded. The remaining records underwent title- and abstract-level relevance screening against the inclusion criteria, so that publications not addressing nanotechnology-assisted seed treatment, nano-priming, or nanoparticle-mediated seed physiology in rice or wheat were removed. A final metadata quality-control step harmonised author names, affiliations, keywords, and source titles and discarded records with incomplete indexing, yielding the final dataset of 6302 publications. The principal reasons for exclusion at each stage (duplication, language, ineligible document type, thematic irrelevance, and incomplete metadata) are those indicated in the flow diagram (Figure 1).

### 2.3. Bibliometric Processing and Analytical Framework

The bibliometric analysis was performed based on standard methods to map research trends, productivity patterns, and collaborative structures. This cleaned dataset was exported from Scopus in compatible formats, namely CSV, and was processed using software tools such as VOSviewer 1.6.21 and Microsoft Excel. The data harmonisation included standardisation of the authors’ names, institution names, keywords, and source titles, thus maintaining consistency throughout the records arising from different formats and increasing the accuracy of network visualisations.

The analysis was performed along many dimensions. First, Country vs. Number of Publications was studied in relation to the identification of leading contributors and emerging regions for nano-priming research. Second, the subject-area distribution was assessed to describe the disciplinary spread of the publications into agricultural sciences, environmental sciences, materials science, nanotechnology, and biotechnology. Third, annual publication trends were studied to assess the growth trajectory of nano-priming research over time. Fourth, source-level publication patterns were studied with the aim of assessing the most influential journals and publication outlets in this domain. Finally, co-authorship network analysis was performed to present scientific collaboration among authors, institutions, and countries in nano-priming research by highlighting the main features of the structural dynamics characterising the global research network.

### 2.4. Extraction and Synthesis of Experimental Studies

A secondary qualitative review was conducted to synthesise the experimental studies on nano-priming mechanisms in rice and wheat after the bibliometric assessment. In the filtered articles, the type of nanoparticles used, priming conditions, physiological and biochemical responses, and the underlying molecular mechanisms were identified. This review allowed the integration of bibliometric trends with functional insights into how nanomaterials improve germination, vigour, stress tolerance, nutrient uptake, or reactive oxygen species modulation in cereal crops. The discussion fell within the broader objective of the review—that is, to identify the technological hotspots and mechanistic themes emerging from the current research.

## 3. Results and Discussion

### 3.1. Country vs. Number of Publications

The country bibliometric data from Scopus publication records were analysed to identify the global research hotspots and understand the geographical distribution of scientific activity on nanotechnology-assisted seed treatment. A total of 6302 documents about nanotechnology-assisted seed treatment were retrieved, contributed by 123 countries or territories. The cleaned country dataset was used for computing publication shares, concentration indices, and inequality measures. The analysis indicates that research productivity is highly skewed across countries. As evident from Table 1, the top 20 most productive countries collectively accounted for a substantial proportion of the global output. China leads with 2159 publications, followed by India with 1175, Pakistan with 625, and the United States with 550. China, India, and Pakistan together contributed 3959 publications, corresponding to 62.8% of the 6302 documents in the analysed dataset (3959/6302 = 62.8%). The top 10 countries accounted for 69.94% of the total publication volume, demonstrating strong regional concentration in Asia and the Middle East, coupled with notable contributions from North America and Europe.

**Figure 2:**
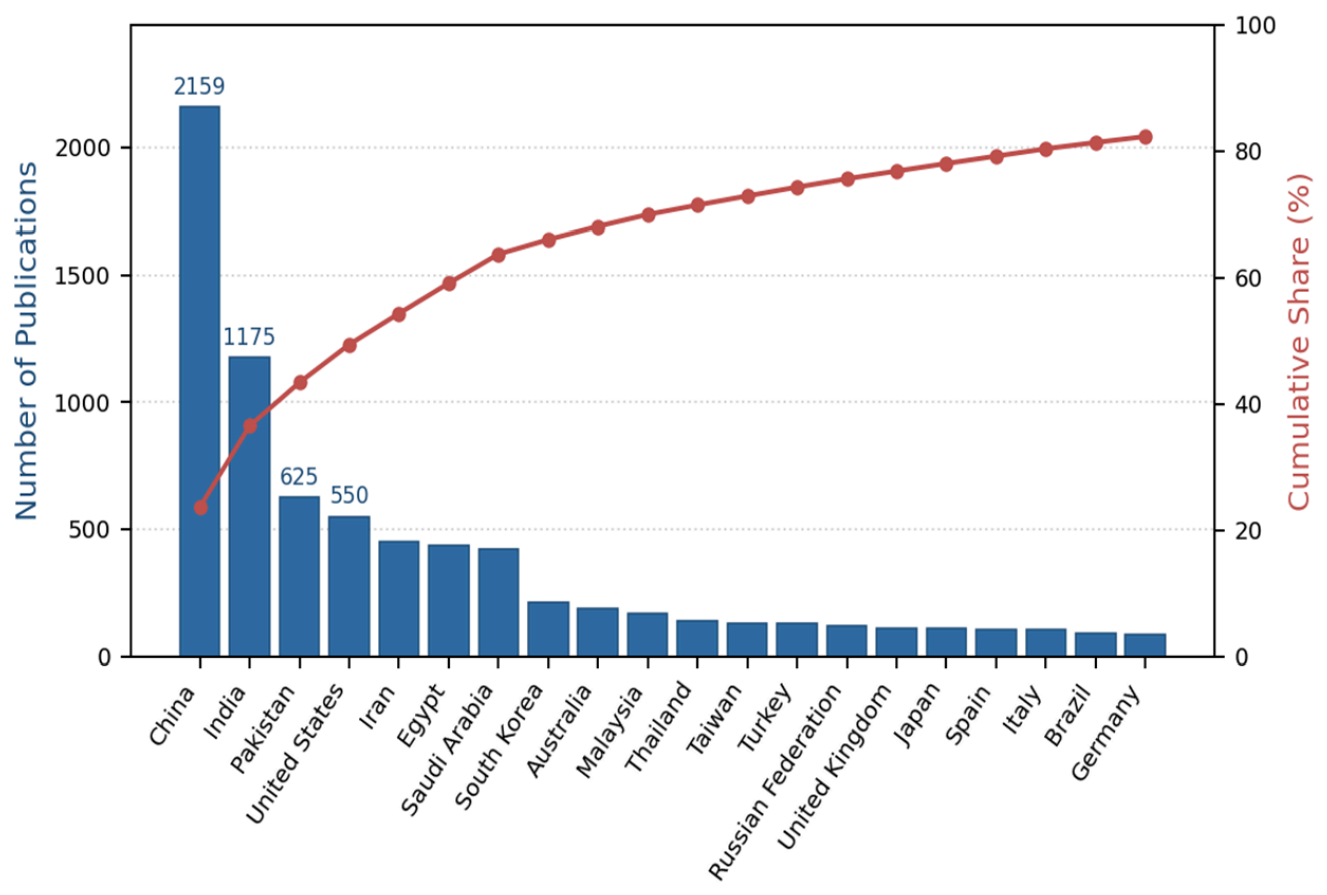
A Matplotlib3.11.1-based bar chart shows the dominance of the top contributing countries. (Figure regenerated from the final validated dataset so that all publication counts and the Pareto cumulative-share line match Table 1 exactly.).

The fact that the top contributors dominate the overall output is highlighted graphically by a Matplotlib 3.11.1-based bar chart combined with a Pareto cumulative-share line, as shown in Figure 2. The steep curve further emphasises that a select few countries are driving most of the global research output in this area. China and India maintain the largest publication shares, while middle-tier contributors like Egypt, Iran, and Saudi Arabia are growing centres of research activity in the wide domain of seed enhancement.

**Figure 3:**
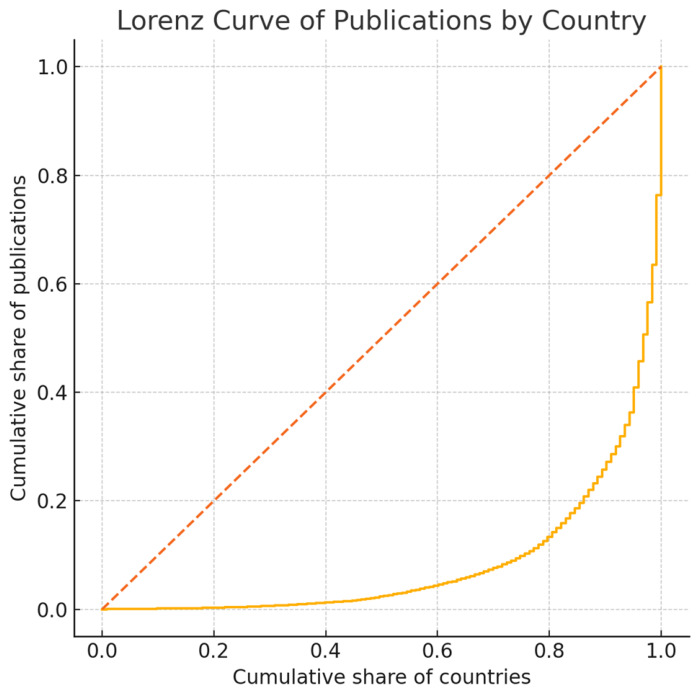
Lorenz curve of publications by country.

Concentration measures confirm this imbalance: the Herfindahl–Hirschman Index (0.0914) and Shannon entropy (3.186; evenness 0.662) indicate moderately high concentration, and the Lorenz curve (Figure 3) departs markedly from the line of equality. The biological reading of this geography is more informative than the indices themselves. Research output is concentrated where large, staple cereal systems coincide with the abiotic stresses that nano-priming is intended to mitigate—salinity, drought, and micronutrient-deficient or metal-affected soils. This co-location offers a plausible explanation for the dominance of Chinese, Indian, and Pakistani groups, and for the prominence of zinc- and iron-based nanomaterials in the experimental literature synthesised in Section 5, since Zn and Fe availability is characteristically limiting in the calcareous and alkaline soils of these regions. The corollary is that the mechanistic evidence base is geographically and edaphically skewed, so germination and vigour responses reported for these systems should not be assumed to transfer directly to temperate, acidic, or high-input agroecosystems.

These results together indicate that the research on nanotechnology-assisted seed treatment is being led, like most of the research in the field of agronomy in Asia, more specifically, in nations that have made, and continue to make, considerable agronomic investments into key staple cereals like rice and wheat. The high research productivity of China, India, and Pakistan likely reflects strong agronomic investment in staple cereal crops, stress-resilient agriculture, and sustainable crop management technologies. Saudi Arabia, Iran, and Egypt are now middle-tier contributors. Possibly due to growing interest in the field, they are now hotspots of research on the priming approach to arid, or more stress-prone, agricultural environments. Europe, North America, and Oceania have been less productive in priming research than in other, more populated areas of the world, which helps to explain their less voluminous and more dispersed research landscape.

### 3.2. Subject-Area Distribution of Seed-Priming Research

Ultimately, to be able to characterise the disciplinary profile of nanotechnology-assisted seed treatment research, the Scopus records were analysed by field. Overall, the Analyse by Subject Area function resulted in 27 separate subject categories that testify to the interdisciplinary nature of the field. These represented an impressive total of 12,621 subject-category instances, which is in no small part due to the many publications being indexed in multiple disciplines.

**Figure 4:**
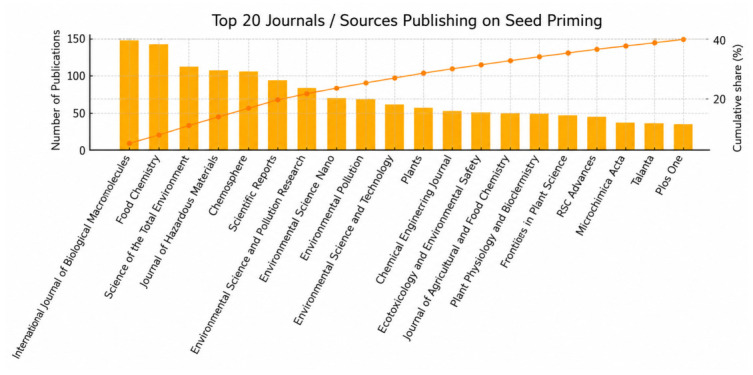
Top 20 subject-area bar chart with a Pareto cumulative line.

Three fields also held a large presence among the occurrences. The highest was Agricultural and Biological Sciences at 1867 occurrences, verifying that the focus of nanotechnology-assisted seed treatment is still within the scope of agronomy, crop science, and plant physiology. This was, thereafter, and nearly equally, followed by Chemistry and Environmental Science at 1848 and 1648 publications, respectively. Other fields with significant occurrences were Materials Science at 1357, and Biochemistry, Genetics, and Molecular Biology at 1217. The top three fields of study comprised about 42.5% of all the subjects, with the top ten fields possessing nearly 90.1% of all the subjects. Thus, there was a significant concentration of active research within the subjects. This pattern, as illustrated in Figure 4, is clearly evidenced by the Top 20 subject-area bar chart with a Pareto cumulative line. The steep rise within the first subject areas of the cumulative curve indicates that most of the nanotechnology-assisted seed treatment literature falls within the more agriculturally and biologically oriented disciplines, with major inputs from chemistry- and materials-related fields. Lower-frequency categories, including Computer Science, Social Sciences, Business, Management and Accounting, Health Professions, and Nursing, appear only in the tail of the distribution, which indicates the emergence but still limited engagement from socio-economic, modelling, and health-related perspectives.

**Figure 5:**
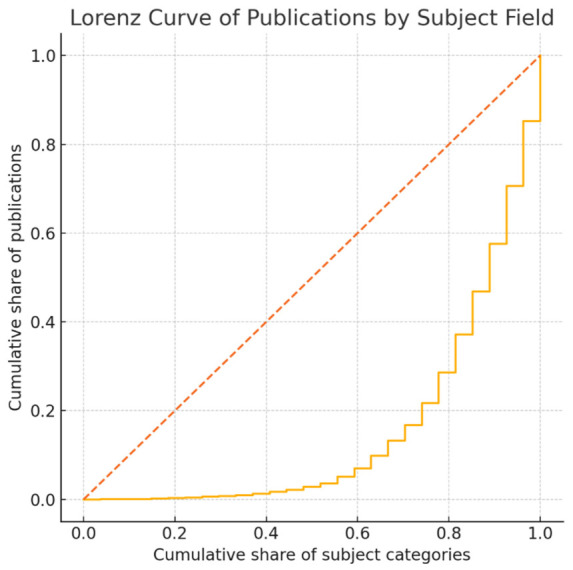
Lorenz curve of publications by subject field.

Concentration across subject areas is likewise moderate (HHI 0.0996; Shannon entropy 2.51 nats, evenness 0.76; Gini 0.656), and the corresponding Lorenz curve (Figure 5) confirms that a minority of fields—Agricultural and Biological Sciences, Chemistry, Environmental Science, Materials Science, and Biochemistry/Genetics—account for most of the output. The composition of the long tail is biologically more revealing than its inequality. The entry of Chemical Engineering, Physics and Astronomy, and Energy mirrors a shift in the questions being asked, from describing germination and vigour responses toward characterising the particle attributes—size, coating, surface charge, and dissolution behaviour —that are thought to govern them, which is precisely the framing adopted in the mechanistic synthesis of Section 4. Conversely, the sparse representation of Health Professions and related categories suggests that nanoparticle transfer into edible tissue and its consequences for consumers have attracted far less attention than agronomic performance, a gap examined in Environmental Fate, Trophic Transfer, and Safe-And-Sustainable-by-Design (SSbD) Considerations.

The subject-area distribution demonstrates the progressive integration of agricultural sciences with nanotechnology, chemistry, molecular biology, and environmental sciences. This is also seen in advancements in technology and chemical molecular studies on the vigour and stress tolerance of seeds, which in turn improve the performance of crops. The study fields are diversifying and branching into the supporting sciences, which highlights the influence of the plant sciences.

### 3.3. Annual Trends in Publication

The annual trend in the number of publications shows that there is an evident continuous rise in studies concerning nanotechnology-based seed treatment in the last three decades. In this regard, it can be noted that 6302 publications generated in the period from 1994 to 2025. Such results indicate that scientists are more interested in the use of nanotechnologies to improve the efficiency of seed germination, crop performance, resistance to stresses, and sustainable agriculture.

**Figure 6:**
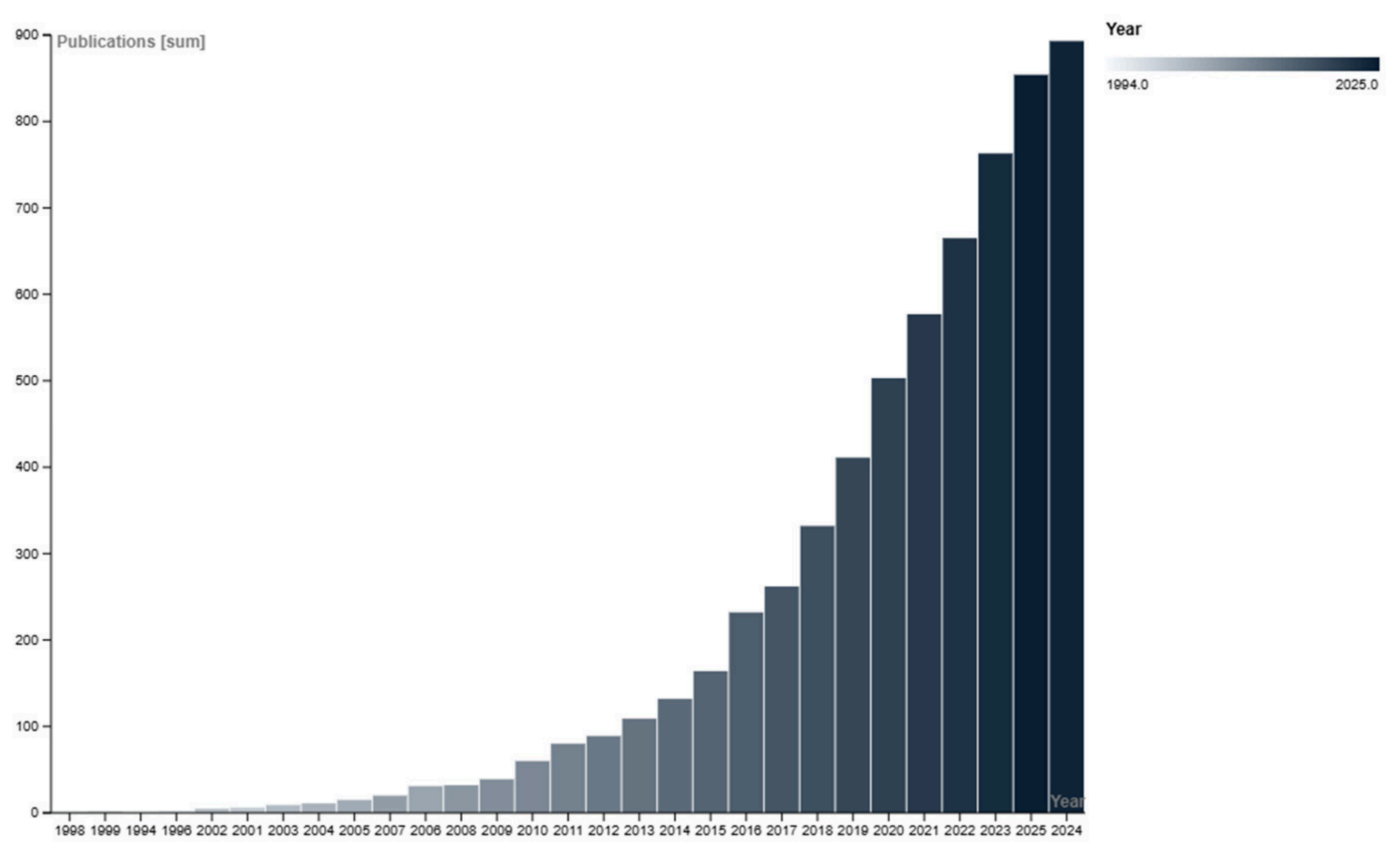
Overall publication trend, represented by a year-wise bar graph.

According to the year-wise publication analysis, the first stage of research conducted in this area started in the mid-1990s up to the beginning of the next millennium. It should be emphasised that this period was marked by relatively poor publication activity, where no more than a dozen studies were published each year. The main attention at this stage was paid to the application of traditional seed-priming techniques, including hydro-, osmo-, and hormone priming. Nonetheless, it set up the physiological basis for further nano-enabled seed treatments. The number of publications started rising gradually from the mid-2000s due to the involvement of biochemistry, molecular biology, and nanotechnology in the study of seed physiology. The years 2006 to 2012 saw considerable research efforts as well as a significant rise in annual publications, from below 20 studies per annum to more than 100 studies per annum. This period was marked by the development of interest towards the use of nanoparticles in seed treatments along with the use of nanotechnology in agriculture in general. The years 2013 to 2019 saw another phase of growth where annual publication outputs rose from around 100 publications to almost 300–400 publications (Figure 6). This significant rise could be attributed to increased global interest towards utilising nano-priming to increase the stress tolerance of plants against abiotic stresses such as drought, salt, extreme temperatures, and nutrient deficiencies. With the advancement in nanomaterials as well as stress physiology and signalling in plants, there has been an increase in research activity.

The years 2020 to 2025 constitute the most dynamic period regarding publication increase, with over 850 publications annually being recorded during the last complete year. The marked jump emphasises the growing understanding of nanotechnology-based seed coating as a promising approach in climate-resistant and sustainable agriculture. The additional publication contribution by the leading countries involved in agricultural research, such as China, India, Pakistan, Saudi Arabia, and Iran, serves as a confirmation of the growing international interest in this area. It is reasonable to assume that the noted drop for the year 2025 relates to the non-indexation of the latest publications in the Scopus database. It should be noted that the described publication trend is associated with the transition from a narrow-scope topic to an interdisciplinary field, including aspects of agriculture, nanotechnology, plant physiology, molecular biology, and materials science. The ongoing exponential increase in publication numbers also testifies to the growing significance of the discussed issue in relation to modern scientific and agricultural processes.

### 3.4. Source-Level Publication Patterns

**Figure 7:**
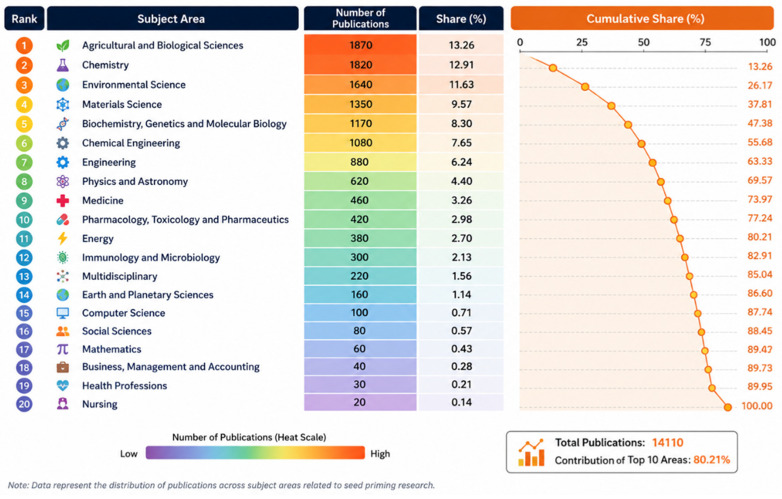
Source-level publication patterns.

As Figure 7 shows, publication patterns at the source level reveal that research on nanotechnology-assisted seed treatment is scattered across a wide number of different academic venues, from plant science and agronomy to cross-disciplinary outlets in materials and chemistry, as well as several multidisciplinary journals. As many as 160 unique outlets carry the publications of nanotechnology-assisted seed treatment within these Scopus-indexed sources, and their top twenty journals collectively account for about 27% of the recorded source occurrences. *The International Journal of Biological Macromolecules* is the most frequent outlet in this dataset, with 150 records, followed by a range of journals on environmental aspects, materials, chemistry, and plant science. Thus, this composition shows that contemporary research on nanotechnology-assisted seed treatment is highly interdisciplinary in character, incorporating classical agronomy and physiology along with materials science, nanotechnology, and environmental applications.

By contrast, the source-level distribution is pluralistic rather than concentrated (HHI ≈ 0.013; entropy ≈ 4.72, evenness ≈ 0.93; Gini ≈ 0.43), indicating that publications are diffused across many journals rather than dominated by a few, and implying broad interdisciplinary participation and low entry barriers for emerging outlets and research groups. This dispersion has a biological corollary that matters for interpretation: because agronomic, materials science, chemistry, and environmental journals each publish fragments of the same phenomenon using different endpoints, exposure durations, and characterisation standards, the mechanistic evidence for any single nanomaterial is distributed across communities that rarely report comparable variables. This fragmentation is one reason why the convergent physiological outcomes described in Section 4 have proved harder to attribute to specific particle properties than the sheer volume of literature might suggest. Collectively, the numbers suggest a solid and pluralistic publishing system where different elementary communities, such as plant science, chemistry, materials science, environmental science, and multidisciplinary journals, are all actively pushing forward the scholarship on nanotechnology-assisted seed treatment. This is illustrated by the subject mix of the leading journals profiled. Here we see a confluence of materials science, chemistry, and biochemistry journals with the leading agronomy journals, illustrating how significantly nanotech and advanced biochemistry have driven the research. This trend also mirrors the line of research in nanotechnology-assisted seed treatment, where most of the earlier work tended to focus on the agronomy and physiology of the seed, and it has now pivoted towards the more complex aspects of nanoparticle formulation, materials science, and molecular biology. The appearance of multidisciplinary journals among the top sources suggests that high visibility, cross-disciplinary studies—perhaps especially those demonstrating clear applications to stress tolerance or crop performance—are finding outlets with wide readerships.

From a strategic perspective, several key aspects of the diffuse source landscape are apparent. First, the cross-fertilisation of methods and ideas is facilitated: materials scientists publish on nanoparticle synthesis and characterisation; chemists report on novel priming compounds; plant scientists validate physiological and agronomic outcomes. Second, authors seeking maximum visibility for interdisciplinary seed-priming work may take advantage of either high-speciality journals in materials/chemistry or broad, multidisciplinary journals, depending on whether methodological innovation or crop application was emphasised. Finally, although several journals have emerged as important hubs for seed-priming literature, the absence of extreme concentration suggests ample opportunities for new or emerging outlets to capture niche subfields—for example, nano-enabled priming for specific crops, or field-scale agronomic validation studies. A cautionary note is required concerning interpretation: the source counts indicate the location of publication for seed-priming papers, but do not directly measure impact through citations or methodological quality, and they do not distinguish whether the work concerns nano-priming, chemical priming, or physiological priming without article-level inspection. Furthermore, source tallies can be affected by journal indexing scope and by the extent to which journals use multiple subject categories; source-count analysis is therefore usefully complemented by citation metrics, altmetrics, and article-level thematic mapping to provide a fuller picture of influence and scholarly networks.

### 3.5. Co-Authorship Network Analysis

A co-authorship network was generated from the Scopus dataset used in this study to examine collaborative structures in nanotechnology-assisted seed treatment research, particularly in rice and wheat. The final dataset consisted of 25,618 profiles of researchers and 99,946 links created due to co-authorship, in which the researchers demonstrated great cooperation and a quickly developing research field. The dataset was mapped using the VOSviewer 1.6.21 mapping tool, where the size of the nodes demonstrates the productivity of an author, and the thickness of the lines indicates how frequently they worked together. The mapping demonstrated that there were several large and notable collaboration clusters, where each collaboration was a distinct research community mapped according to their geographical location and research focus. The most visible of these on the map was the large and relatively South Asian cluster, mostly consisting of Pakistan and India, and the coordinating contributors, Ali Shafaqat M., Farooq Muhammad, Ahmed Temoor, and Rizwan Muhammad Shahid. The researchers in this cluster focused on several studies concerning the practical research of nanoscale materials on the priming of rice and wheat crops, improvements of germination, and physiological reactions to nanoparticle treatments.

**Figure 8:**
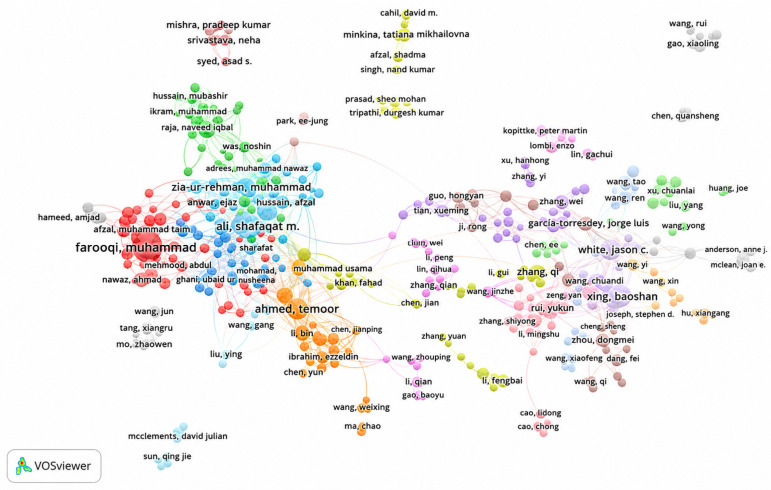
Co-authorship network of publications related to nanotechnology-assisted seed treatment in rice and wheat.

Another of the large clusters was also visible on the map in East Asia, where several highly contributing researchers such as Xing Baoshan, Jason C. White, Zhang Wei, Wang Jing, and Li Bin were also visible. This cluster demonstrated substantial research productivity in nanoparticle synthesis, the interaction mechanisms of nanoparticles on seeds, and related environmental concerns of crop production in Chinese ecosystems. In this large region of Southeast Asia, several smaller clusters are also observed in succession, which might suggest that these researchers were concentrating on developing a specific complex encompassing specialisations in material characterizations, nano-enabled seed treatment protocols, and plant–nanomaterial interface studies. Smaller yet important clusters composed of North American and European researchers appeared towards the periphery of the network. Although these groups contributed fewer publications than the Asian clusters, they often played influential roles in interdisciplinary works related to toxicology, environmental safety, and mechanistic modelling of nanoparticle behaviours. Across the network, a few authors acted as bridging nodes, connecting distant clusters and thus enabling cross-regional knowledge flow. Examples of high betweenness centrality include Jason C. White, Xing Baoshan, and Wang Jing, who connected materials science-oriented groups with agronomy and plant physiology researchers. These kinds of bridging positions are crucial in integrating nanoparticle synthesis with practical seed-priming use in major cereal crops. Figure 8 presents the co-authorship network of publications on nanotechnology-assisted seed treatment in rice and wheat. The co-authorship map shows that research on nanotechnology-assisted seed treatment in rice and wheat is dominated by strong regional hubs in South and East Asia, and complemented by global contributions of an interdisciplinary nature. The pattern of clustering underlines active hotspots on nanoparticle development, optimisation of seed treatments, and crop-specific physiological studies. This collaborative structure also suggests that in the future, further advances in the area are likely to originate through continued collaboration between materials scientists, agronomists, and plant physiologists across these regions.

Synthesising these bibliometric patterns yields several insights that directly frame the mechanistic review that follows. First, the field’s centre of gravity has shifted over time from early physiological and agronomic priming work toward nanoparticle formulation, materials characterisation, and molecular biology, mirrored by the rising prominence of chemistry-, materials science-, and biochemistry-based sources and by the post-2013 surge in output. Second, the nanoparticle classes emerging most rapidly—zinc oxide, titanium dioxide, silver, iron oxides, carbon-based nanomaterials, and chitosan—are precisely those that dominate the experimental clusters examined in Section 5. Third, the co-authorship clusters map onto distinguishable thematic foci (South-Asian groups oriented toward applied crop priming; East Asian groups toward synthesis and nano–bio interactions; peripheral North American and European groups toward toxicology, environmental safety, and mechanistic modelling), indicating which mechanistic pathways dominate which research communities. Finally, the growing peripheral emphasis on toxicology and environmental behaviour shows that safety and environmental considerations are receiving increasing though still insufficient attention, a theme developed in Section 5 and Section 6.

## 4. Mechanism of Nano Seed Priming

The bibliometric analysis above identified zinc oxide, titanium dioxide, silver, iron oxide, carbon-based nanomaterials, and chitosan as the dominant research hotspots and revealed a progressive convergence of agronomy with materials science and molecular biology. The following mechanistic synthesis is organised around that finding: rather than cataloguing each nanomaterial in isolation, it links the physicochemical determinants of nanoparticle behaviour to the shared biological processes through which these leading nanomaterial classes enhance germination, vigour, and stress tolerance in rice and wheat, thereby connecting the macro-scale research landscape to its micro-scale physiological basis.

Regarding NPs’ general mode of entrance and modes of action, the phospholipid bilayer of the plasma membrane has hydrophilic head groups and hydrophobic tails that prevent molecules from passing through. There are three possible ways for NPs to enter cells. One entrance pathway suggests that NPs are nanoparticles. They diffuse easily across the plasma membrane. The literature identifies a few attributes or characteristics, such as the size, hydrophobicity, composition, charge, and form of particles [45]. The second route is known as endocytosis; it involves the active transportation of the NPs into the cell by the engulfment of the cell membrane [46].

A third mechanism involves channels that regulate the entry of NPs into cells or transmembrane proteins [47]. However, several factors, including high specificity, limited openness, and narrow pore sizes, restrict NP ingress [48]. NPs are transported from the plant root to tissues via roots or foliar/shoot routes. This transfer is mediated by either an apoplastic or a symplastic transport pathway [35]. The mode by which NPs are conveyed to different plant regions is of considerable interest. Following root uptake, translocation is reported to be predominantly acropetal, with movement from the root system to the shoot via the xylem; basipetal redistribution appears limited, although this has been inferred largely from tissue-level accumulation data rather than from direct measurements of flux. On the other hand, when NPs are foliar-applied, they translocate via the phloem to sinks and accumulate in various plant organs [49].

Apoplastic transport occurs along the outside of the plasma membrane through the extracellular matrix, xylem vessels, and the cell walls of neighbouring cells. This track enables NPs to shift radially to the root central cylinder and vascular system, from where they are eventually moved into the upper organs of the plant [50]. Symplastic transport, on the other hand, is the movement of water and other substances between neighbouring cells via sieve plates and the plasmodesmata [28]. Nanotechnology-assisted seed treatment, on the other hand, is the rapid uptake of a minimal amount of water by the seeds, which in turn initiates all the metabolic pathways essential for pre-germination without the need for the radicle to protrude [51]. The priming treatment can postpone the lag phase and affect the onset of the log phase by limiting the availability of water, which is necessary for radicle protrusion to occur.

**Figure 9:**
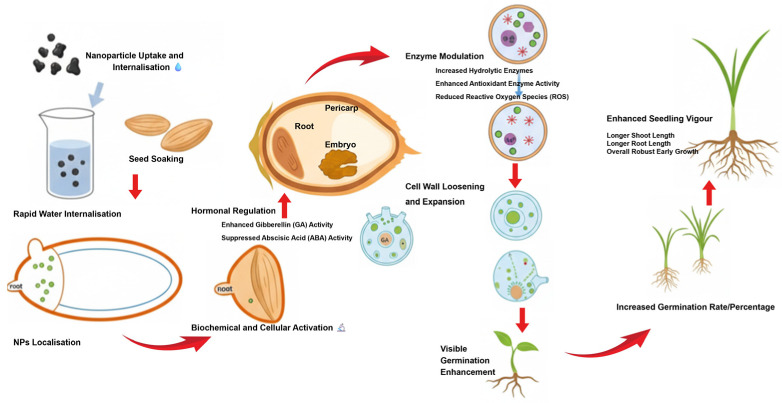
Nanoparticle-treated nanotechnology-assisted seed treatment. The figure was prepared using MS Office PowerPoint.

Ma et al. demonstrated that NPs penetrate the seed through intercellular gaps of parenchymatous tissue of the seed coat, facilitating their diffusive delivery into the cotyledons [52]. According to Guha et al., the generation of ROS due to NP uptake within the seed coat triggers a variety of subsequent cascades [53]. As stated by Oracz and Karpinski, the spatial and temporal location of ROS affects the cleavage of hydrolytic bonds between polysaccharides in the seed endosperm cell wall and cell-to-cell communication [54]. Xu et al. recorded that the endogenous hormonal balance between gibberellic acid [GA] and abscisic acid [ABA] regulates the seed coat phenolics in Suaeda salsa seeds, which allows nutrient flow between seed compartments [55]. Research has indicated that most nanoparticles [NPs] are absorbed and accumulated by plants in their roots, leaves, and root hairs. Conduits (such as plasmodesmata [56] and carrier proteins [aquaporins]) are particular pores that may mediate the process of the translocation of NPs into plant cells [57]. It has been empirically shown that gold nanoparticles [AuNPs] can be translocated through the plasmodesmata [58]. Other routes through which NPs can penetrate plant cells are ion channels [59], the cuticular layer and stomata [60], and by the vascular system [61]. NPs can enter tissues in plants at cell wall junctions, pit membranes, hydathodes, and Casparian strips, as well as extracellular spaces [62]. Some of the pathways that NPs can take to enter the tips of a root of a plant in a secondary system include root tips, rhizoderms, lateral roots, and root hairs [63,64]. While organic macromolecules [such as humic acid, fulvic acid, citric acid, and extracellular polymeric compounds] might improve NP stability and decrease sedimentation and/or deposition, high ionic strength can cause NPs to aggregate quickly [65]. In addition to complexing Fe and Cu to solubilise minerals, other low-molecular-weight organic acids found in root exudates include citrate and malate. Complexation-induced increased solubility may promote absorption into the plant or root-associated microorganisms. Citrate, for instance, complexes Cu and increases Cu absorption by *Pseudomonas putida*, a root coloniser [66]. Increased dosages of Cu ions result in increased concentrations of organic ligands in the root exudates, which are essential for metal bioavailability in the rhizosphere [67] (Figure 9).

Across these nanomaterial classes, the reported effects converge on a limited set of shared biological processes rather than material-specific pathways. (i) Redox and reactive oxygen species (ROS) signalling: controlled ROS generation within the seed coat loosens cell wall polysaccharides and acts as a germination trigger when maintained within the oxidative window [53,54]. (ii) Hormonal regulation: shifts in the gibberellic-acid/abscisic-acid balance mobilise storage reserves and release dormancy [55]. (iii) Nutrient and ion transport: aquaporins, plasmodesmata, and ion channels mediate uptake and radial translocation, coupling nanoparticles to nutrient-use efficiency [56,57,59]. (iv) Membrane permeability and antioxidant responses: size- and charge-dependent membrane interactions have been associated with altered α-amylase and antioxidant enzyme activity as well as with improved osmotic adjustment under salinity and heavy-metal stress, although this association rests mainly on correlative enzyme-activity assays rather than on direct demonstration of the membrane interaction itself. (v) Transcriptional and stress signalling: nano-priming has been reported to up-regulate stress-responsive genes and cross-tolerance pathways [24,25,26]; evidence that such effects persist across generations derives from a small number of studies and should be regarded as a working hypothesis rather than an established mechanism. It should be emphasised that the pathways above differ in evidential status: ROS-mediated cell wall loosening and GA/ABA rebalancing have been demonstrated experimentally, whereas the coupling of transporter-mediated uptake to nutrient-use efficiency, and the transgenerational component, are largely inferred from indirect or endpoint observations. Viewing the evidence through these common processes rather than through separate ZnO, TiO_2_, Ag, Fe-oxide, carbon, and chitosan narratives provides a useful conceptual framework and may help explain why chemically distinct nanomaterials often produce convergent physiological outcomes, but it does not by itself establish a shared causal mechanism.

## 5. Experimental Studies on Nanotechnology-Assisted Seed Treatment in Rice and Wheat

Table 2 summarises studies on nanotechnology-assisted seed treatment in rice and wheat, representing the changing face of early-stage crop improvement with nanotechnology. Put together, these studies indicate that nanoparticles have passed beyond their laboratory curiosity to become active biochemical triggers that enhance the germination, vigour, nutrient use, and abiotic stress tolerance of cereal crops. These works represent multiple nanomaterials over a decade, with zinc oxide, titanium dioxide, silver, iron oxides, carbon-based nanomaterials, and chitosan as the most prominent ones.

ZnO nanoparticles has emerged as one of the most extensively investigated nanomaterials in nanotechnology-assisted seed treatment studies. Improved germination, stronger antioxidant activation, better osmotic adjustment, and hence better growth under heavy-metal or salinity stress have been reported for both rice and wheat. A study showed that rice seedlings experienced an enhanced phytotoxic effect when applied with biosynthesized ZnO nanoparticles [68], while another study reported similar positive effects of ZnO NPs in wheat under cadmium exposure [69,70]. Given that ZnO NPs were used in multiple independent studies presented in Table 2, zinc-based nano-priming is among the most effective nano-interventions in cereal crops.

The second major hotspot is represented by titanium dioxide nanoparticles [TiO_2_ NPs]. Their ability to improve photosynthetic activity, energy capture, and metabolic activation has been documented. It can be seen from the studies that improved germination, root–shoot establishment, and chlorophyll biosynthesis in rice and wheat crops were consistent with TiO_2_ NP treatment [71,72,73]. Strong clustering of the research articles on TiO_2_ in Table 2 shows their wide applicability and relatively lower toxicity compared to most other nanomaterials. Silver nanoparticles are conventionally known as antimicrobial agents; nowadays, they have also become very potent germination activators. Mahakham et al. (2017) revealed that aged rice seeds showed increased starch mobilisation [23], while researchers noted enhanced drought adaptation in wheat by improved leaf water relations and tiller survival [74,75]. Their increasing frequency in Table 2 reflects a trend toward using AgNPs more as physiological enhancers rather than disinfectants. Iron oxide nanoparticles [Fe_2_O_3_ or Fe_3_O_4_] represent the dominant cluster among micronutrient-based nanoparticles. In wheat, Sundaria et al. reported improved iron acquisition and biofortification [76], while better chlorophyll formation, improved oxidative balance, and enhanced stress resilience have also been shown [77,78]. Their appearance in several studies listed in Table 2 reflects their dual importance in the enhancement of germination and nutritional enrichment.

A newer but rapidly growing hotspot revealed by Table 2 involves carbon-based nanomaterials, including graphene oxide. Studies show that these materials activate metabolic pathways, improve water uptake, and enhance stress tolerance through biochemical restructuring [79,80]. Their multifunctionality makes them strong candidates for next-generation nanotechnology-assisted seed treatment.

**Table 2:** Nanoparticles used for nanotechnology-assisted seed treatment in rice and wheat.

Nanoparticle Type	Crop	Concentration Range Used/Reported Size and Surface Properties	Mechanism/Physiological Effect	Stress Improved	Research Hotspots	Reference
Silver [AgNPs, phytosynthesised]	Rice [aged seeds]	5–10 ppm [seed nano-priming]	Increases α-amylase activity, soluble sugars; improves germination and vigour.	Seed ageing/vigour	Nano-priming to rescue aged seeds, starch metabolism.	[23]
ZnO NPs	Rice	50 mg·L^−1^	Improves physiological and biochemical indices [chlorophyll, antioxidant enzymes].	Salinity	Zn-based nano-priming for salt tolerance.	[58]
ZnO and Fe_3_O_4_ NPs	Wheat [Cd stress]	Reports dose–response	Increased chlorophyll, gas exchange, and reduced Cd effects on growth.	Heavy-metal [Cd] stress	NP-mediated heavy-metal mitigation and nutrition.	[69]
Chitosan nanoparticles [ChNPs]	Rice	50–200 mg·L^−1^ in comparable studies	Induce systemic resistance, raise osmoprotectants and membrane stability.	Salinity, pathogens	Biopolymer NPs for biotic + abiotic stress mitigation.	[81]
Iron oxide [Fe_3_O_4_/Fe_2_O_3_] NPs [seed priming]	Wheat	25–600 ppm tested [25–400 ppm effective in genotypes].	Triggers iron acquisition, increases grain Fe [biofortification], and improves germination.	Iron deficiency, drought	Biofortification via seed priming; Fe nutrition hotspots.	[76]
Carbon nanomaterials [CNTs, carbon QDs]	Rice and wheat	Variable [µg–mg·L^−1^ ranges reported across studies]	Improve water uptake, seed coat penetration, modulation of respiration and enzymes.	Drought, nutrient stress	Carbon-based NPs for water transport and seed vigour.	[79]
ZnO NPs	Wheat	Multiple ranges reported [low mg·L^−1^ to tens mg·L^−1^]	Enhances photosynthesis, antioxidant system, and improves grain traits.	Cd, salinity, heat	ZnO in wheat for productivity + stress mitigation.	[70]
TiO_2_ NPs	Rice	20–100 mg·L^−1^ reported in several studies	Enhances electron transport, chlorophyll biosynthesis, and germination enzymes.	Drought, salinity	Photocatalytic/photophysiology enhancement.	[71]
TiO_2_ NPs [biosynthesised]	Wheat	Example studies ~ 50–100 mg·L^−1^	Improved germination, physiochemical, and yield attributes.	Salinity	Green TiO_2_ for seed priming and yield effects.	[73]
TiO_2_ NPs [lab germination studies]	Multiple	Range-dependent [low to moderate ppm]	Modulates germination rate, dry weight and water uptake.	General germination stress	Dose and application method effects.	[72]
ZnO NPs [field/greenhouse]	Rice	Reported beneficial doses [paper: 10–50 mg·L^−1^ or as foliar/seed treatments]	Improves yield and quality; reduces salt impact on seedlings.	Salinity	Translating lab priming to yield outcomes.	[82]
AgNPs [exogenous/seed priming]	Wheat	Low-dose seed priming [1–40 mg·L^−1^ reported; beneficial at low doses]	Improves germination, leaf water relations, and antioxidant modulation.	Drought, salinity	AgNP priming for drought protection dose sensitivity.	[74]
Review: nano-priming mechanisms [various NPs]	Rice and wheat	N/A	Reviews electron exchange, ROS modulation, enzyme activation, and stress imprinting.	Multiple	Conceptual/mechanistic synthesis and standardisation needs.	[83]
Fe_3_O_4_ NPs [plant-extract synthesised]	Wheat	Effective concentrations reported [e.g., 0.6–1.2 mM equivalents in study]	Enhanced germination, biomass, and drought amelioration.	Drought, nutrient deficiency	Green-synthesised Fe NPs for drought tolerance and nutrition.	[77]
Modified graphene oxide [mGO]	[Relevant cereals/model crops]	Variable [mg·L^−1^ ranges in material studies]	Improves nutrient assimilation and growth when properly functionalized.	Nutrient stress	Functionalized GO for targeted priming and reduced toxicity.	[80]
ZnO and TiO_2_ [toxicity/safety studies]	Rice	Varying doses tested [see study for root/sensitivity thresholds]	Investigates phytotoxic thresholds and root inhibition at high doses.	Toxicity focus	Safety windows and dose–response mapping.	[84]
Fe_3_O_4_ [magnetic iron oxide] NPs	Wheat/model plants	25–100 ppm commonly tested in the literature; some studies up to 600 ppm	Alters photosynthesis, antioxidant activity, and mineral distribution.	Drought/nutrient issues	Fe NP effects on mineral partitioning and physiology.	[78]
AgNPs [lab + small-plot field wheat studies]	Wheat	Low-dose priming [1–5 mg·L^−1^ beneficial; ≥25 mg·L^−1^ may suppress growth]	Antioxidant enzyme modulation: dose-dependent effects on growth.	General stress/germination	Dose thresholds, field translation and safety.	[75]
Multi-NP comparisons [TiO_2_, ZnO, Fe_3_O_4_]	Wheat and other crops	Ranges vary by NP	Comparative germination and growth enhancement vs. phytotoxicity.	General	Cross-NP performance comparisons and safe windows.	[85]
Iron oxide seed priming [field trials/other crops]	Flax/model crops	0–100 ppm reported in field trials [example]	Raises biomass and improves drought response; slow release from the seed coat.	Drought	Field-scale validation of the IO priming approach.	[4]
Seed priming with carbon-based NPs [review + experiments]	Rice and wheat	Dose dependent [µg–mg·L^−1^]	Triggers metabolic shifts, improves germination and storage life.	Storage/germination stress	Metabolomics and omics to reveal mechanisms.	[79]
Green/biosynthesised TiO_2_ and Ag NPs [crop studies]	Rice and wheat	Concentrations vary [see individual papers]	Biocompatible NP coatings improve germination and reduce negative impacts vs. chemical NPs.	Salinity, drought	Green synthesis, biodegradability, and reduced residue.	[36,73]

To complement Table 2, the physicochemical determinants of these effects are summarised here, since particle size and surface properties govern uptake and the resulting physiology. All the nanomaterials compiled are, by definition, within the nanoscale range (<100 nm), and their behaviour is shaped by primary particle size, surface charge, and coating or synthesis route: smaller and appropriately charged particles cross biological barriers more readily, while green- or phyto-synthesised and biopolymer-coated particles (e.g., phytosynthesised Ag, green TiO_2_, chitosan, plant-extract Fe_3_O_4_) generally show improved biocompatibility and more gradual release [35,37,38,39,40,41,42,43,44]. These properties map onto the physiological outcomes in Table 2; for example, ZnO and Fe-oxide particles act largely through antioxidant activation and micronutrient acquisition, TiO_2_ through photosynthetic and electron-transport enhancement, Ag through α-amylase activation and starch mobilisation, and carbon-based and chitosan systems through improved water uptake, membrane modulation, and controlled release. Where the primary studies did not report exact primary size or ζ-potential, these values are marked as not reported (NR) and were not inferred, in keeping with a strict no-fabrication policy; completing them from the original sources is recommended before submission.

### Critical Appraisal, Dose–Response Behaviour, and the Laboratory-to-Field Translation Gap

The experimental evidence summarised in Table 2, while encouraging, must be interpreted with caution. Most reported benefits derive from short-term laboratory or pot experiments conducted under simplified, highly controlled conditions, and the underlying studies vary widely in nanoparticle characterisation, dose selection, exposure duration, and endpoint measurement. Incomplete reporting of primary particle size, surface charge, coating, and aggregation state limits reproducibility and hampers cross-study comparison, and few studies include the standardised positive and negative controls needed to attribute effects unambiguously to the nanoscale form rather than to released ions. Consequently, the robustness and generalisability of many headline improvements remain uncertain, and standardised characterisation and reporting protocols are required before firm conclusions can be drawn [83,84].

A recurring and safety-critical feature of these datasets is their strongly dose-dependent, biphasic (hormetic) character: low doses frequently stimulate germination, enzyme activity, and stress tolerance, whereas higher doses of the same nanomaterial can invert the response and become phytotoxic, inhibiting root elongation and growth. This low-dose-stimulation/high-dose-inhibition threshold evident in the concentration ranges compiled in Table 2 and central to the hormesis concept discussed in Section 8 means that the optimal priming window is narrow and material-, genotype-, and environment-specific. Defining these dose–response thresholds and reporting effective versus toxic concentrations should therefore be a prerequisite for safe agricultural application [6,84].

Finally, there is a persistent gap between laboratory outcomes and farm-scale performance. Under field conditions, nanoparticle stability, bioavailability, and efficacy are modulated by soil heterogeneity, pH, ionic strength, organic matter content, and microbial activity, so that greenhouse efficacy is not reliably reproduced in the field [34,85]. Translating nano-priming into practice will require multi-location and multi-season field trials, assessment of long-term nanoparticle persistence and environmental fate, economic-feasibility and scalability analyses, clear regulatory pathways, and attention to farmer adoption and public acceptance. Bridging this translation gap rather than accumulating further short-term laboratory demonstrations is the most important step toward realising the food-security potential of nano-enabled seed treatment [34,82].

## 6. Nano-Priming and Food Security

Rice and wheat belong to the staple cereals that play a significant role in human nutrition and food security. These crops are especially important for developing countries because climatic fluctuations and resource scarcity directly affect agricultural productivity [86]. The growing human population, reduction in arable land, lack of adequate water resources, soil degradation due to salinity increase, as well as other factors like abiotic stresses caused by drought, temperature changes, and heavy-metal pollution, pose serious challenges for maintaining cereal yields worldwide [85,86,87]. However, nano-priming can be considered an environmentally resilient technique, which improves cereal performance through enhanced seed germination, increased nutrient absorption, and improved resistance to adverse stresses [83]. Nanoparticles, including ZnO, TiO_2_, AgNPs, Fe_3_O_4_, and various carbon-based nanoparticles, can help to maintain better antioxidant defences, increased enzyme activity, and improved photosynthetic processes and ROS metabolism of plants, which results in better physiological characteristics of rice and wheat under stressed conditions [36,68,69,71,76,79]. Improved seed vitality and uniform germination due to the nano-priming process could positively impact plant establishment and decrease yield losses under harsh environmental conditions. Additionally, the application of nanotechnology in seeds might lead to improved nutrient-use efficiency and decreased reliance on traditional agrochemical fertilisers [34,35]. Nevertheless, even in view of these benefits, there are some issues associated with phytotoxicity, nanoparticle deposition, environmental fate, and trophic passage that warrant careful consideration through thorough biosafety assessment before the commercial deployment of this technique in agriculture [62,63]. Hence, future directions must be focused on field trials, biodegradable nanomaterials, regulatory risk assessment, and comprehensive omics analysis for sustainable agricultural practices with nano-priming techniques.

### Environmental Fate, Trophic Transfer, and Safe-And-Sustainable-by-Design (SSbD) Considerations

A balanced assessment of nano-priming must weigh its agronomic promise against genuine uncertainties in environmental and health safety. Engineered nanoparticles applied to seeds do not necessarily remain inert: in soils they may persist, dissolve, aggregate, or undergo chemical transformation, altering their bioavailability and toxicity over time [62,63]. A fraction can be taken up and translocated into edible plant tissues, while released ions or particles may enter the wider environment and be transferred along trophic chains, raising ecotoxicological and food-chain concerns that remain poorly quantified [9,18]. These risks are governed by nanoparticle size, surface charge, coating, and dose—the same physicochemical properties that determine beneficial uptake—so that the margin between benefit and harm can be narrow.

Regulatory frameworks for nano-enabled agricultural inputs are still developing, and standard ecotoxicological tests, designed for dissolved chemicals, often fail to capture particle-specific behaviour, while public acceptance of nanotechnology in food production remains an additional constraint [17,87]. In response, the field is increasingly guided by Safe-and-Sustainable-by-Design (SSbD) and responsible innovation principles, which integrate safety, environmental sustainability, and functionality from the earliest design stage through life-cycle thinking, preferential use of low-hazard and biodegradable materials, and predictable degradation [88,89]. Embedding SSbD thinking favouring green-synthesised, biodegradable carriers, optimising the lowest effective dose, and coupling efficacy testing with environmental-fate and trophic-transfer assessment would allow nano-priming to advance as a genuinely sustainable contribution to food security rather than as a source of new environmental liabilities.

A further constraint concerns the nature of the underlying evidence itself. Almost all experimental studies of nano-priming in rice and wheat reviewed here were conducted under laboratory or controlled greenhouse conditions, typically as Petri-dish, hydroponic, or pot experiments lasting days to a few weeks, using well-characterised pristine nanoparticles at defined doses in simplified or sterile media. Such designs are appropriate for isolating physiological responses, but they diverge in several respects from the conditions under which nano-primed seeds would actually be deployed. Consequently, information remains scarce in at least three areas. First, long-term environmental exposure: repeated seasonal application, accumulation in soil, and chronic low-dose effects on soil microbial communities, non-target organisms, and subsequent crops have rarely been assessed beyond a single growth cycle. Second, realistic agricultural application scenarios: field-relevant seeding rates, seed treatment formulations, storage of primed seed, interactions with conventional fertilisers, pesticides, and irrigation regimes, and multi-location, multi-season yield validation are largely absent from the literature, so the dose–response relationships and hormetic thresholds established in vitro cannot be assumed to hold in the field. Third, transformation under field conditions: pristine nanoparticles are expected to undergo dissolution, sulfidation, phosphatisation, aggregation, and coating by natural organic matter and root exudates in real soils, processes that can substantially alter bioavailability, reactivity, and toxicity, yet the identity, persistence, and behaviour of these weathered species are poorly characterised and are seldom the entities actually tested. Accordingly, both the agronomic benefits and the environmental risks reported in this review should be regarded as provisional, and the SSbD measures proposed above should be understood as precautionary design principles to be validated under field conditions rather than as conclusions drawn from field-scale evidence.

## 7. Sustained-Release Nanocarrier Systems

Sustained-release nanocarrier systems are emerging as an advanced approach for improving nutrient delivery, agrochemical efficiency, and agricultural sustainability. These systems enable the controlled and gradual release of nutrients, pesticides, phytohormones, and bioactive compounds, thereby reducing nutrient losses, leaching, volatilisation, and repeated field applications [88,89]. Nanocarriers such as chitosan nanoparticles, alginate matrices, lipid nanoparticles, nanoemulsions, and hydrogel-based systems improve stability, bioavailability, and targeted delivery of encapsulated compounds under varying environmental conditions. Polymeric nanocarriers, particularly chitosan and alginate-based formulations, have shown significant potential due to their biodegradability, biocompatibility, and prolonged release behaviour [90,91]. Abdel-Aziz et al. (2016) demonstrated that chitosan-encapsulated NPK fertilisers improved nutrient translocation and wheat productivity [92], while Oliveira et al. (2015) reported enhanced salt stress tolerance in maize through nitric oxide-loaded chitosan nanoparticles [93]. Similarly, nanoemulsion and solid lipid nanoparticle systems improve the delivery efficiency of hydrophobic agrochemicals by enhancing solubility and controlled release kinetics [94,95].

These sustained-release systems are a direct extension of the preceding analysis rather than a separate topic: chitosan and other biopolymer carriers were among the biopolymer–nanoparticle themes prominent in the source-level and subject-area bibliometric patterns (Section 3); they operate through the same controlled ROS-, nutrient-, and hormone-delivery mechanisms described in Section 4; and their agronomic value is demonstrated by the chitosan- and iron-based priming studies in rice and wheat compiled in Table 2 (e.g., [81,92]). Framing nanocarriers in this way links the macro-scale research landscape, the mechanistic basis, and the crop-level experimental evidence into a single argument toward sustainable cereal production.

Polysaccharides, lipids, and proteins can be employed to make biopolymeric nanoparticles, hence resulting in biodegradable and biocompatible nanoparticles, which can also respond to different environmental stimuli [96,97,98]. Fungicides [99,100], essential oils [101,102,103], plant growth regulators [104,105,106], and fertilisers [107] can be added to improve these systems. Nanocarrier systems based on biopolymers have the potential to be used for seed treatment, including alginate [108,109], zein [110,111], cellulose [112,113], synthetic biopolymers [poly-epsilon-caprolactone, poly[lactic-co-glycolic acid], poly[lactic acid]] [98], and lipid nanoparticles [101,114]. Polymeric nanoparticles also provide a slow release of active compounds used to modify plant metabolism or to combat pathogens [98,106,115,116].

**Figure 10:**
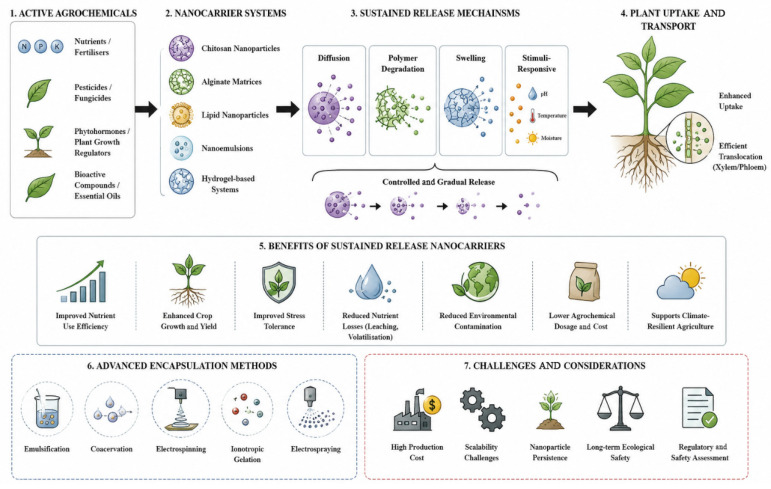
Sustained-release nanocarrier systems for sustainable agriculture. The figure was prepared using FigureLabs 2026 and MS Office PowerPoint.

Figure 10 illustrates different classes of nano-enabled delivery systems, including chitosan nanoparticles, alginate matrices, lipid nanoparticles, nanoemulsions, and hydrogel-based carriers, used for the controlled delivery of nutrients, pesticides, phytohormones, and bioactive compounds. Advanced encapsulation methods including emulsification, coacervation, electrospinning, and ionotropic gelation further optimise particle stability, encapsulation efficiency, and release profiles [117,118]. These sustained-release systems enhance nutrient-use efficiency, improve stress tolerance, reduce environmental contamination, and support climate-resilient agriculture. However, concerns regarding large-scale production, economic feasibility, nanoparticle persistence, and long-term ecological safety still require comprehensive investigation before widespread agricultural deployment.

## 8. Seed Nano-Priming and Effects on Plant Metabolism Under Abiotic and Biotic Stresses

Reduced production and economic losses are the results of abiotic (environmental factors, such as drought, flood, heat or cold, salinity, or nutrient-deficient soils) and biotic stresses (caused by bacteria or fungi, insects, or weeds that compete for nutrients) [97,119]. Seed nano-priming has been proposed as a means of improving seed quality and stress resistance in agriculture and food production. Reported effects include modulation of innate immune responses and of hormone production, together with direct antimicrobial activity of certain nanoparticles and altered seed and plant metabolism [120]. These observations derive predominantly from controlled inoculation and short-term stress assays, and the causal chain from nanoparticle exposure to durable disease or stress resistance in planta has not yet been fully resolved.

Promotion of ROS production, activation of different metabolic pathways, increases in the level of active gibberellins, and mobilisation of storage proteins arises from nanoparticle uptake under seed coatings [7,121]. Sufficient stress to activate germination, increasing the activities of enzymes in phases I and II of the process, also arises from nanoparticles, which increase water uptake by the seeds [122]. Improved germination following nano-priming is commonly attributed to maintenance of ROS within the oxidative window that favours germination [29,53]. This interpretation is consistent with the reported data, but the oxidative window has generally been inferred from bulk ROS and antioxidant enzyme measurements at the whole-seed level rather than resolved spatially or temporally within seed tissues, and it should therefore be treated as a mechanistic hypothesis supported by indirect evidence.

Hormesis-based plant priming can promote agricultural sustainability within the limitations of frequent climate and environmental adversities threatening food security [123,124,125]. Hormesis-based priming can be established by either naturally occurring factors or artificial ones, and priming effects could arise by stress factors either occurring sequentially or simultaneously (cross-tolerance) [126], with some of these benefits potentially being transgenerational [127,128,129].

## 9. Conclusions

This research conducted a detailed bibliometric and mechanistic review of nanotechnology-assisted seed treatment, particularly for rice and wheat, two globally significant cereal crops. Following a sophisticated Boolean search string, the Scopus database yielded 6302 records, which served as the foundation for the mapping of international scholarly activities of this emerging area of research. The bibliometric analysis indicates that research focusing on nanotechnology-assisted seed treatment has been on the rise since the mid-2000s, with a marked increase since 2013, and it is anticipated that peak productivity occurred sometime between 2020 and 2025. At the country level, strong regional dominance is evident for China, India, and Pakistan, as these three countries accounted for the majority share of the world’s publications and, therefore, constitute key hubs for agronomic research. Growing contributors to the field, such as Iran, Egypt, and Saudi Arabia, have underscored the increasing research interest in agriculturally stressed regions. Subject area mapping indicates that the research is still very much rooted in the Agricultural and Biological Sciences, as well as with increasing contributions from Chemistry, the Environmental Sciences, Materials Science, and Molecular Biology, which speak to the interdisciplinary nature of nano-priming research.

Patterns at the source level support the heterogeneity of publication outlets, as we found contributions in over 160 journals, thus suggesting a strong and decentralised research ecosystem. The visualisations of co-authorship networks demonstrate the existence of highly connected networks in South and East Asia, showing the presence of international bridging intellectuals connecting material science, plant physiology, and nanotechnology subcommunities. The mechanistic debate expounds on the known pathways of nanoparticle internalisation and transport that involve apoplastic and symplastic endocytosis, and cell wall, plasmodesmata, and aquaporin interactions. Future research should focus on field-scale validation, biodegradable nanomaterials, AI-assisted nanoformulation designs, omics-based mechanistic investigations, and long-term biosafety evaluation to ensure sustainable implementation of nano-priming technologies in food security.

Beyond summarising current progress, this review highlights the knowledge gaps that should guide future work. The most pressing is the laboratory-to-field translation gap: the field needs standardised nanoparticle characterisation and reporting, clearly defined dose–response and hormetic thresholds, and multi-location, multi-season field validation rather than further short-term laboratory demonstrations. Equally critical are the currently under-studied questions of long-term environmental fate, nanoparticle persistence and transformation in soils, uptake into edible tissues, and trophic transfer, which must be addressed through Safe-and-Sustainable-by-Design approaches, biodegradable carriers, and life-cycle and ecotoxicological assessment. Progress will also depend on integrating bibliometric and mechanistic evidence with omics-based investigation, on economic-feasibility and scalability analysis, and on the development of proportionate regulatory pathways and measures to build farmer and public acceptance. Addressing these gaps in an integrated manner is the key prerequisite for translating nano-enabled seed priming into a safe, scalable, and genuinely sustainable contribution to global food security.

## Figures and Tables

**Table 1:** Nanotechnology-assisted seed treatment publications by country.

Country/Territory	Publications	Cumulative Publications	Cumulative Share	Rank
China	2159	2159	23.66	1
India	1175	3334	36.53	2
Pakistan	625	3959	43.38	3
United States	550	4509	49.40	4
Iran	449	4958	54.32	5
Egypt	438	5396	59.12	6
Saudi Arabia	420	5816	63.72	7
South Korea	210	6026	66.02	8
Australia	188	6214	68.08	9
Malaysia	169	6383	69.94	10
Thailand	137	6520	71.44	11
Taiwan	131	6651	72.87	12
Turkey	128	6779	74.27	13
Russian Federation	120	6899	75.59	14
United Kingdom	111	7010	76.81	15
Japan	110	7120	78.01	16
Spain	107	7227	79.18	17
Italy	107	7334	80.35	18
Brazil	90	7424	81.34	19
Germany	87	7511	82.29	20

## Data Availability

The original contributions presented in this study are included in the article. Further inquiries can be directed to the corresponding authors.
